# The *KCNJ11* E23K Polymorphism and Progression of Glycaemia in Southern Chinese: A Long-Term Prospective Study

**DOI:** 10.1371/journal.pone.0028598

**Published:** 2011-12-05

**Authors:** Chloe Y. Y. Cheung, Annette W. K. Tso, Bernard M. Y. Cheung, Aimin Xu, Carol H. Y. Fong, K. L. Ong, Lawrence S. C. Law, Nelson M. S. Wat, Edward D. Janus, Pak C. Sham, Karen S. L. Lam

**Affiliations:** 1 Department of Medicine, Li Ka Shing Faculty of Medicine, The University of Hong Kong, Pokfulam, Hong Kong; 2 Department of Psychiatry, Li Ka Shing Faculty of Medicine, The University of Hong Kong, Pokfulam, Hong Kong; 3 Research Centre of Heart, Brain, Hormone and Healthy Aging, Li Ka Shing Faculty of Medicine, The University of Hong Kong, Pokfulam, Hong Kong; 4 Department of Medicine, University of Melbourne, Western Hospital, Footscray, Victoria, Australia; 5 Genome Research Centre, Li Ka Shing Faculty of Medicine, The University of Hong Kong, Pokfulam, Hong Kong; Universita Magna-Graecia di Catanzaro, Italy

## Abstract

**Context:**

The *KCNJ11* E23K variant is associated with type 2 diabetes mellitus (T2DM) in cross-sectional studies, but conflicting findings have been reported from prospective studies.

**Objective:**

This study aimed to evaluate whether the E23K variant could predict glycaemic progression in a Southern Chinese population.

**Methods/Principal Findings:**

We performed a long-term prospective study on 1912 subjects from the Hong Kong Cardiovascular Risk Factors Prevalence Study (CRISPS). The *KCNJ11* E23K variant was associated with the progression to prediabetes after a median interval of 12 years on multinomial logistic regression analysis, even after adjustment for traditional risk factors (OR 1.29, P_age, sex, BMI and fasting plasma glucose [FPG] adjusted_  = 0.02). Based on Cox proportional hazard regression analysis, the E23K variant also predicted incident prediabetes (HR 1.18, P_age, sex, BMI and FPG adjusted_  = 0.021). However, E23K was not associated with the progression to T2DM in either multinomial or Cox regression analysis, and the association of E23K with glycaemic progression to either prediabetes or T2DM was significant only in unadjusted Cox regression analysis (P = 0.039). In a meta-analysis of eight prospective studies including our own, involving 15680 subjects, the E23K variant was associated with incident T2DM (fixed effect: OR 1.10, P = 4×10^−3^; random effect: OR 1.11, P = 0.035).

**Conclusions:**

Our study has provided supporting evidence for the role of the E23K variant in glycaemic progression in Chinese, with its effect being more evident in the early stage of T2DM, as the subjects progressed from normal glucose tolerance to prediabetes.

## Introduction

Type 2 diabetes mellitus (T2DM) is a complex metabolic disorder characterized by impaired insulin secretion and insulin resistance. The pancreatic beta-cell adenosine triphosphate (ATP)-sensitive potassium channel (K_ATP_ channel), regulates insulin secretion through the coupling of glucose metabolism to membrane electrical activity [1]. The K_ATP_ channel consists of the inwardly-rectifying potassium channel pore-forming (Kir6.2) subunits and the sulphonylurea receptor 1 (SUR1) subunits, encoded by the inwardly-rectifying potassium channel subfamily J member 11 (*KCNJ11*) gene and the ATP-binding cassette subfamily C member 8 (*ABCC8*) gene, respectively. Based on its potential function, *KCNJ11* was identified as one of the T2DM susceptibility genes through the candidate gene study approach [2]. A non-synonymous variant at codon 23 of the *KCNJ11* gene, which causes a glutamic acid to lysine (E23K; rs5219) substitution at the N-terminal, has been shown to be associated with T2DM across various ethnic groups [3], [4], including Chinese[5], [6], in cross-sectional studies. *In vitro* cell-based studies suggested that the E23K polymorphism causes a reduction in the sensitivity of the Kir6.2 subunit towards ATP, thereby leading to the inhibition of insulin secretion [7]. The functional significance of the E23K variant has also been confirmed *in vivo*, based on its association with the disposition index, an insulin secretion index which taken into consideration the insulin sensitivity of an individual [8]. More recently, the association of the E23K variant and a different *KCNJ11* variant rs5215 [I337V; in strong linkage disequilibrium (LD) with E23K] with T2DM have been confirmed in several genome-wide association studies [9], [10], [11], generating renewed interests in its potential role as a genetic marker for T2DM development.

Conflicting results have been obtained, however, in prospective studies on the association between the development of T2DM and the E23K variant or its closely linked variant I337V. The population-based Hisayama Japanese prospective study, which considered only subjects with normal glucose tolerance (NGT) at baseline, was the first to demonstrate that the E23K variant could predict the development of T2DM [12]. Subsequently, the DESIR Prospective Study reported significant association of this variant with incident T2DM [13]. Moreover, the Nurses' Health Study of the United States (US) [14] and the EPIC-Potsdam prospective study of Germany [8] also described the association of this variant with T2DM development in female subjects. In contrast, two earlier studies involving only subjects with impaired glucose tolerance (IGT) at baseline from the US Diabetes Prevention Program [15] and the Finnish Diabetes Prevention Study (DPS) [16], the E23K polymorphism was not associated with T2DM development [12]. A recent prospective study in Shanghai Chinese, which examined the effect of the *KCNJ11* I337V on T2DM development, failed to demonstrate any association [17]. Given these divergent reports, it is important to further examine, prospectively, the clinical relevance of the E23K variant on the progression of glycaemia. The current study aimed to evaluate, in a long-term prospective study, whether the E23K variant could predict the progression of glycaemia in Southern Chinese.

## Methods

### Ethics statement

All participants were studied after written informed consent, and the study protocol was approved by the Ethics Committee of the University of Hong Kong.

### Subjects

**Figure 1:**
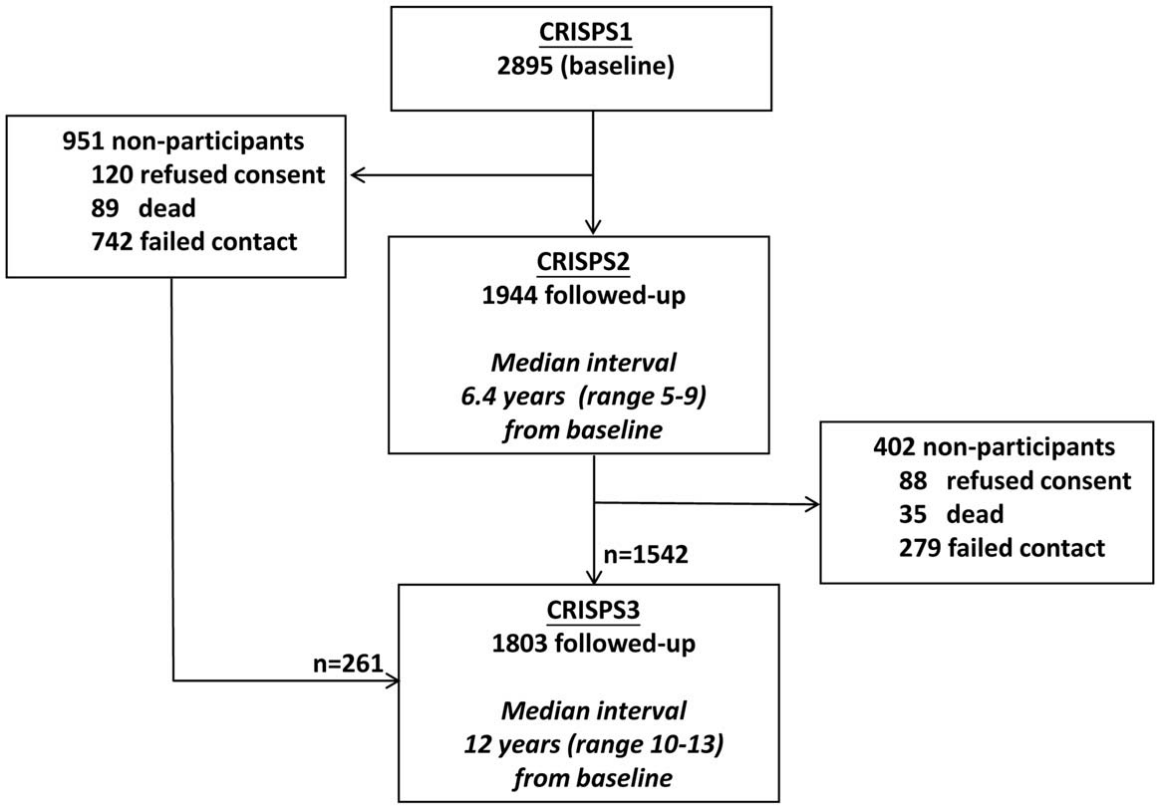
Flow of CRISPS study subjects.

The Hong Kong Cardiovascular Risk Factors Prevalence Study (CRISPS) is a population-based longitudinal study of cardiovascular risk factors, in which unrelated Southern Chinese subjects were recruited from the general population via random telephone numbers in Hong Kong. Details of the CRISPS cohort were previously described [18]. Briefly, in 1995–96 (baseline study), 2895 Hong Kong Chinese were invited to undergo a comprehensive assessment of cardiovascular risks, including a 75 g oral glucose tolerance test (OGTT). In 2000–2004, 1944 subjects returned for follow-up study (CRISPS2) [19]. In 2005–2008, 1803 subjects participated in the latest 12-year follow-up study (CRISPS3) (Figure 1). Subjects were classified as having NGT, IGT, impaired fasting glucose (IFG) or T2DM according to the World Health Organisation (WHO) 1998 diagnostic criteria [20]. Prediabetes was defined as having either IGT or IFG. Subjects who were non-diabetic at baseline but had been receiving anti-glycaemic drugs by CRISPS2 or CRISPS3 were classified as T2DM at the respective follow-up. The inclusion criteria for subjects involved in each analysis are as follow:

- **Progression to prediabetes or T2DM by the end of the 12-year follow-up.** Progression to prediabetes: subjects with NGT at baseline who had progressed to IGT or IFG by CRISPS3 (12-year follow-up). Progression to T2DM: non-diabetic subjects (NGT/IGT/IFG) at baseline who had developed T2DM by CRISPS3.No progression: non-diabetic subjects (NGT/IGT/IFG) at baseline whose glycaemic status remained unchanged by CRISPS3 (i.e. persistent NGT/IGT/IFG).
- **Prediction of incident prediabetes.** Incident prediabetes: subjects with NGT at baseline who had developed IGT or IFG by CRISPS2 or CRISPS3. Persistent NGT: subjects with NGT at baseline who remained NGT at subsequent follow-up visit(s).
- **Prediction of incident T2DM.** Incident T2DM: subjects who were non-diabetic (NGT/IGT/IFG) at baseline and had converted to T2DM by CRISPS2 or CRISPS3. Non-diabetic: subjects who were non-diabetic (NGT/IGT/IFG) at baseline and remained as non-diabetic at subsequent follow-up visit(s).
- **Prediction of glycaemic progression.** Glycaemic progression: subjects with NGT at baseline who had developed IGT, IFG or T2DM by CRISPS2 or CRISPS3; and subjects who were IGT/IFG at baseline who had converted to T2DM by CRISPS2 or CRISPS3. Persistent NGT or persistent IGT/IFG: subjects who were NGT or IGT/IFG at baseline whose glycaemic status remained unchanged in subsequent follow-up visit(s).

### Anthropometric and biochemical measurements

The anthropometric (including body mass index [BMI] and waist circumference [WC]) and biochemical measurements (including fasting plasma glucose [FPG], 2 hr post-OGTT glucose [2 hrG] and fasting plasma insulin) were measured as previously described [19]. The insulin resistance index based on the homeostasis model assessment (HOMA-IR) was calculated as (FPG [mmol/l] x fasting plasma insulin [mIU/l]/22.5) and homeostasis model assessment of beta-cell function (HOMA-beta) was calculated as (20 x fasting plasma insulin [mIU/l]/FPG [mmol/l]-3.5) [21].

### Genetic Analyses

The E23K variant was genotyped by the TaqMan Pre-designed SNP Genotyping Assay (Assay ID:C__1594245_10; Applied Biosystems, Foster City, CA, USA). Polymerase chain reactions were performed in the GeneAmp®PCR System 9700 thermal cycler according to the manufacturer's protocols, and assay products were analyzed using Applied Biosystems PRISM® 7000 Sequence Detection System for fluorescence intensity detection. Hardy-Weinberg equilibrium (HWE) was examined by the DeFinetti program (http://ihg2.helmholtz-muenchen.de/cgi-bin/hw/hwa1.pl). The successful genotyping call rate was 100% and concordance rate was 97.5%.

### Meta-analysis and meta-regression analysis

We searched the PubMed database to identify relevant articles related to the association of the *KCNJ11* E23K or its closely linked variant I337V with incident T2DM. We used the terms “*KCNJ11*” and “Type 2 diabetes” as key words to search for the related articles. Publications were included in the current study if they investigated, in a prospective design, the association of the E23K or I337V variants with incident T2DM and provided sufficient data (e.g. ethnicity, odds ratio [OR] and genotype counts) to allow us to perform the analysis. For publications in which the crude OR was not provided, OR under per-allele comparison was calculated for analysis. Two publications [13], [15] were excluded since the available information was insufficient for us to calculate the ORs. We used the Cochran's χ^2^ test (Q-test) to examine the heterogeneity between studies and a P-value of less than 0.05 was considered as significant. We also performed a meta-regression analysis to evaluate whether particular covariates, including the total sample size, ethnicity and follow-up time, could explain the heterogeneity effects between different studies. The meta-analysis was conducted by using the Comprehensive meta-analysis version 2.0 software. The meta-regression analysis was performed by SPSS (Version 16.0; Chicago, Illinois).

### Statistical Analysis

All statistical analyses were performed with SPSS (Version 16.0; Chicago, Illinois). Variables that did not follow a normal distribution were natural-logarithmically-transformed before analysis. Multinomial logistic regression and Cox proportional hazards regression (Cox regression) analyses were employed.

- **Multinomial logistic regression.** Multinomial logistic regression was used to analyse the association of the E23K variant in three categories of subjects simultaneously: (1) progression to T2DM (NGT/IGT/IFG at baseline ->T2DM by CRISPS3), (2) progression to prediabetes (NGT at baseline -> IGT/IFG by CRISPS3) and (3) persistent NGT or persistent IGT/IFG.
- **Cox regression.** The Cox regression analyses (time-to-event analyses) were also performed to evaluate the predictive value of this variant with incident prediabetes, incident T2DM and glycaemic progression. Subject's glycaemic statuses at all follow-up visit(s) (CRISPS2 and/or CRISPS3) were considered.

Baseline variables that were significantly different (P<0.05) between different groups or were biologically likely to affect glycaemic status were included in the analyses with multiple adjustments for confounding factors. Three models which involved different sets of confounding factors were used in the multiple adjustment analyses. Multiple adjustments were made for age, sex, BMI and FPG in model 1; age, sex, BMI, FPG and 2 hrG in model 2; and age, sex, BMI and HOMA-IR in model 3. All associations were tested under the additive model. Power calculations for the Cox regression analyses were performed using the PASS 11 software. A two-tailed P-value of less than 0.05 was considered as statistically significant.

## Results

The current prospective study, included all subjects from the CRISPS cohort who were non-diabetic at baseline and in whom the E23K variant was successfully genotyped using stored DNA samples (n = 1912). The frequency of the risk (K) allele in the CRISPS cohort (0.34) was comparable with those previously reported in Chinese [5], [6], [17]. The genotype distribution of this variant was in HWE in all analyses (all HWE P-values P >0.05, ranging from 0.07 to 0.94).

### Progression to prediabetes or T2DM by the end of the 12-year follow-up

Table 1 shows the baseline clinical characteristics of 209 subjects who had progressed to prediabetes (NGT at baseline -> IGT/IFG by CRISPS3), 198 subjects who showed progression to T2DM (NGT/IGT/IFG at baseline -> T2DM by CRISPS3), and 1185 subjects who showed no progression in glycaemic status by the end of the 12-year study. Compared to those who showed no progression, subjects who progressed to prediabetes or T2DM consisted of more men (P = 0.019), were older, had greater BMI, WC, FPG and 2 hrG levels during OGTT, fasting insulin (all p<0.001) and HOMA-IR (P = 0.002). Differences in the level of HOMA-beta, family history of diabetes and exercise (>30 minutes per week) were not statistically significant (P>0.05).

**Table 1:** Baseline clinical characteristics of subjects who showed no progression, progression to prediabetes and progression to T2DM at the end of 12-year.

Baseline Parameters	No progression	Progression to prediabetes	Progression to T2DM	Overall P-value
Number	1185	209	198	-
Sex (Male%)	44.8	52.2	53.5	0.019
Age (year)	42.6±10.9	45.5±11.2^**^	48.7±11.5^***^	<0.001
BMI (kg/m^2^)	23.4±3.3	24.7±3.4^***^	26.6±3.7^***^	<0.001
WC (cm)	Male:80.7±8.3	Male:84.3±8.7^***^	Male:88.2±8.4^***^	<0.001
	Female:73.2±8.4	Female:76.6±8.5^**^	Female:81.9±9.5^***^	<0.001
FPG (mmol/l)	5.0±0.4	5.1±0.4	5.4±0.5^***^	<0.001
2 hrG (mmol/l)	5.9±1.5	6.1±1.1	7.7±1.8^***^	<0.001
Fasting insulin ( µU/ml)**^a^**	4.5(3.0–6.8)	5.3(3.4–7.8)*	6.6(4.4–10.5)^***^	<0.001
HOMA-IR**^a^**	1.0(0.7–1.5)	1.2(0.8–1.8)*	1.6(1.1–2.5)^***^	0.002
HOMA-beta **^a^**	60.0(42.1–86.2)	63.7(42.2–91.9)	65.0(44.4–91.1)	0.393
T2DM family history(%)	17.1	19.7	20.4	0.404
Excercise (>30minutes per week)	43	44.5	38.4	0.399

Bonferroni Post Hoc test was applied for multiple comparisons. No progression as reference.

*P<0.05; ** P<0.01; *** P<0.001.

Data as mean±standard deviation or median with interquartile range. **^a^** Natural-log-transformed before analysis. NGT: Normal glucose tolerance; IGT: Impaired glucose tolerance; IFG: Impaired fasting glucose; BMI: Body mass index; WC: Waist circumference; FPG: Fasting plama glucose; 2 hrG: 2 hr post-OGTT glucose; HOMA-IR: homeostasis model assessment index for insulin resistance; HOMA-beta: homeostasis model assessment index for beta-cell function.

In the multinomial logistic regression analysis, the E23K was significantly associated with progression to prediabetes (unadjusted P = 0.011; OR[95%CI]:1.32[1.07–1.63]) but was not associated with progression to T2DM (unadjusted P = 0.961; OR[95%CI]:1.01[0.81–1.26]) when compared to subjects who showed no progression (Table 2). The possible association of the E23K variant with progression to prediabetes was further analysed with adjustments for different sets of confounding factors (Table 3). In all three models, the E23K variant remained an independent predictor for progression to prediabetes (Model 1: P = 0.020, OR[95%CI]:1.29[1.04–1.60]; Model 2: P = 0.022, OR[95%CI]: 1.28[1.04–1.59]; and Model 3: P = 0.016, OR[95%CI]:1.31[1.05–1.62]). As previous studies have suggested that the E23K variant might affect insulin secretion [7], [8], association of the E23K variant with an index for insulin secretion, HOMA-beta, was examined by the linear regression analysis. However, the E23K variant showed no significant association with HOMA-beta (P = 0.573; beta[95%CI]:0.01[−0.03, 0.06]).

**Table 2:** Multinomial logistic regression analysis showing the association of the *KCNJ11* E23K variant with progression to prediabetes and progression to T2DM at the end of the 12-year study.

		Genotype count						
	n	KK	EK	EE	HWE P-value	EAF	OR (95%CI)	unadjusted P
Progression to T2DM	198	23	86	89	0.749	0.333	1.01 (0.81–1.26)	0.961
Progression to prediabetes	209	35	96	78	0.556	0.397	**1.32 (1.07**–**1.63)**	**0.011**
No progression	1185	137	513	535	0.407	0.332	1.00 (Ref)	-

HWE: Hardy-Weinberg Equilibrium; EAF: Effect allele frequency; n: Number; OR: Odds ratio; CI: Confidence interval; P: P-value.

**Table 3:** Multiple multinomial logistic regression showing the association of the *KCNJ11* E23K variant with progression to prediabetes and progression to T2DM, adjusted for confounding factors.

	Model 1^a^		Model 2^b^		Model 3^c^	
	OR (95%CI)	Adjusted P	OR (95%CI)	Adjusted P	OR (95%CI)	Adjusted P
Progression to T2DM	1.01 (0.79–1.28)	0.958	0.94 (0.72–1.21)	0.613	1.00 (0.78–1.27)	0.981
Progression to prediabetes	**1.29 (1.04**–**1.60)**	**0.020**	**1.28 (1.04**–**1.59)**	**0.022**	**1.31 (1.05**–**1.62)**	**0.016**
No progression	1.00 (Ref)	-	1.00 (Ref)	-	1.00 (Ref)	-

a Model 1: Multiple adjustments made for age, sex, BMI and fasting glucose.

b Model 2: Multiple adjustments made for age, sex, BMI, fasting glucose and 2-hr glucose.

c Model 3: Multiple adjustments made for age, sex, BMI and HOMA-IR.

OR: Odds ratio; CI: Confidence interval; P: P-value; Ref: Reference.

### Prediction of incident prediabetes, incident T2DM or glycaemic progression in the long-term prospective study

We further examined the predictive value of the E23K variant with incident prediabetes (NGT at baseline -> IGT/IFG by CRISPS2 or CRISPS3) by using the Cox regression analysis. 412 incident prediabetes cases were compared with 1048 subjects with persistent NGT when censored at the end of the study. The E23K variant was significantly associated with incident prediabetes (unadjusted P = 2×10^−3^, HR [95%CI]:1.25[1.09–1.44]) (Table 4). With adjustment for age, sex, BMI and FPG, the E23K variant remained as an independent predictor for incident prediabetes (Adjusted P = 0.021, HR[95%CI]:1.18[1.02–1.35]) and showed a marginal association with incident prediabetes after adjustment for age, sex, BMI and HOMA-IR (Adjusted P = 0.051, HR[95%CI]:1.15[1.00–1.33]) (Table 5). We also investigated the association of the E23K variant with incident T2DM (NGT/IGT/IFG at baseline -> T2DM by CRISPS2 or CRISPS3) by comparing 226 incident T2DM cases with 1686 subjects who were non-diabetic when censored at the end of the study. However, the E23K variant did not show a significant association with incident T2DM (unadjusted P = 0.655, HR[95%CI]:0.96[0.79–1.16]). Finally, the Cox regression analysis was performed for glycaemic progression (NGT at baseline -> IGT/IFG/T2DM by CRISPS2 or CRISP3; and IGT/IFG at baseline -> T2DM by CRISP2 or CRISPS3) which involved both incident prediabetes and incident T2DM case subjects. A total of 638 subjects who showed glycaemic progression were compared with 1274 subjects who showed no progression in glycaemic status and the E23K variant showed a significant association (unadjusted P = 0.039, HR[95%CI]:1.13[1.01–1.26]). However, the association with glycaemic progression became insignificant after adjustment for the confounding factors (P >0.05 in all 3 models).

**Table 4:** Cox regression analysis showing the association the *KCNJ11* E23K variant with incident prediabetes.

		Genotype count						
	n	KK	EK	EE	HWE P-value	EAF	HR (95%CI)	unadjusted P
Incident prediabetes	412	69	177	166	0.067	0.382	**1.25 (1.09**–**1.44)**	**2**×**10^−3^**
Persistent NGT	1048	106	453	489	0.942	0.317	1.00(Ref)	-

HWE: Hardy-Weinberg Equilibrium; EAF: Effect allele frequency; n: Number; HR: Hazards ratio; CI: Confidence interval; P: P-value; Ref: Reference.

**Table 5:** Multiple Cox regression analyses showing factors independently associated with incident prediabetes.

	Incident prediabetes					
Risk factors	Model 1^b^		Model 2^c^		Model 3^d^	
	HR (95%CI)	Adjusted P	HR (95%CI)	Adjusted P	HR (95%CI)	Adjusted P
E23K (KK)	**1.18 (1.02**–**1.35)**	**0.021**	**1.15 (1.00**–**1.32)**	**0.048**	1.15 (1.00–1.33)	0.051
Age (years)	1.03 (1.03–1.04)	<0.001	1.03 (1.02–1.04)	<0.001	1.04 (1.03–1.05)	<0.001
Sex (Male)	1.15 (0.95–1.40)	0.157	1.01 (0.83–1.23)	0.922	1.03 (0.84–1.25)	0.793
BMI (kg/m^2^)	1.07 (1.04–1.10)	<0.001	1.05 (1.02–1.08)	0.001	1.07 (1.04–1.10)	<0.001
FPG (mmol/l)	1.95 (1.51–2.53)	<0.001	1.65 (1.26–2.16)	<0.001	-	-
2 hrG (mmol/l)	-	-	1.51 (1.37–1.67)	<0.001	-	-
HOMA-IR^a^	-	-	-	-	1.22 (1.02–1.45)	0.029

a Natural-log-transformed before analysis.

b Model 1: Multiple adjustments made for age, sex, BMI and fasting glucose.

c Model 2: Multiple adjustments made for age, sex, BMI, fasting glucose and 2-hr glucose.

d Model 3: Multiple adjustments made for age, sex, BMI and HOMA-IR.

BMI: Body mass index; FPG: Fasting plama glucose; 2 hrG: 2 hr post-OGTT glucose; HOMA-IR: homeostasis model assessment index for insulin resistance; HR: Hazard ratio; CI: Confidence interval; P: P-value.

### Meta-analysis and meta-regression analysis

**Figure 2:**
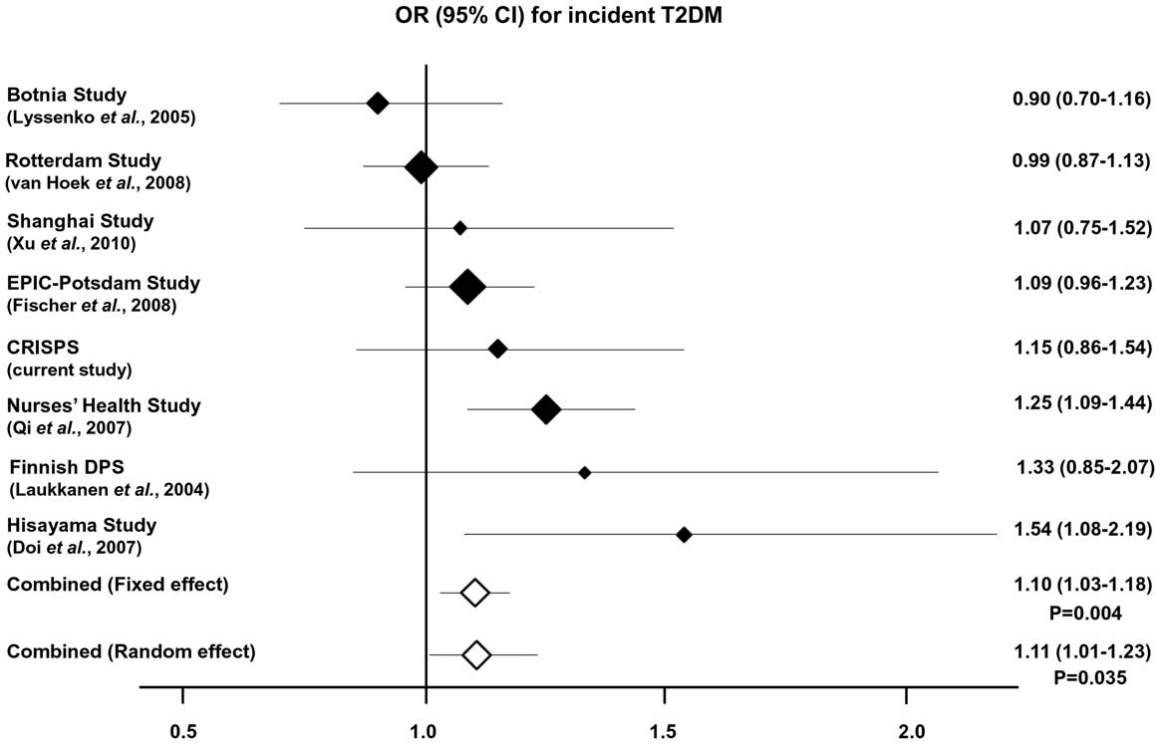
Forest Plot. Meta-analysis of eight prospective studies on the association of *KCNJ11* E23K variant with incident T2DM. The shaded diamonds represent the individual OR of each study. The size of these shaded diamonds were proportional to the study weighting in the meta-analysis. The un-shaded diamonds indicate the combined ORs calculated under the fixed and random effect models. The 95% confidence intervals are demonstrated by the horizontal lines.

To comprehensively investigate the possible role of the E23K variant in the prediction of T2DM, we conducted a meta-analysis by combining our data with those from previous publications. We combined our data with seven previous publications which examined the E23K [8], [12], [14], [16], [22], [23] or the strongly linked I337V variant [17] in a prospective design. The association of the E23K variant with T2DM incidence was investigated in a total of 2425 incident T2DM cases and 13255 controls. A significant association was observed between the E23K variant and incident T2DM (Fixed effect: P = 4×10^−3^; OR(95%CI):[1.10(1.03–1.18)]; Random effect: P = 0.035; OR(95%CI):[1.11(1.01–1.23)]). Figure 2 shows the forest plot of the association between the E23K variant and incident T2DM. There was subtle evidence of heterogeneity between studies, although the Q-test was statistically insignificant (P = 0.087). By using meta-regression analysis, we found that the follow-up time (P = 8×10^−4^) and total sample size (P = 4×10^−3^) were the covariates that could explain the heterogeneity between studies.

## Discussion

In the current study, the *KCNJ11* E23K variant was shown to be associated with glycaemic progression, in the development of prediabetes from NGT. However, we failed to demonstrate the significant association of this variant with T2DM incidence. While previous studies of the E23K variant in Chinese have focused mostly on cross-sectional analyses [5], [6], our well-established CRISPS cohort, with 12-year prospective data, has allowed us to study the contribution of this genetic variant to glycaemic progression in a prospective setting. Case-control studies are useful for the initial screening for susceptibility variants. On the other hand, prospective studies are more helpful for substantiating the clinical relevance of the identified variants.

Our data from the multinomial logistic regression showed that the E23K variant was significantly associated with the progression to prediabetes but not with the progression to T2DM at the end of 12-year study. Results from the Cox regression models also showed that E23K could predict incident prediabetes but could not predict incident T2DM. These findings would support the hypothesis previously raised by Florez *et al.* that the E23K polymorphism may exert its effect early in the course of T2DM, from NGT to prediabetes, rather than influencing the progression from prediabetes to T2DM. They were unable to show the significant association of the E23K variant with T2DM development in a large prospective study which involved 3534 subjects who had IGT at baseline [15]. In fact, previous studies have demonstrated the presence of impaired insulin secretion in the early stage of T2DM [24].

On the other hand, our observation that the E23K variant only showed a significant association with the development of prediabetes but not with that of T2DM could have been due to a lack of power in the current study. With the modest effect size of the E23K variant, we have insufficient power to detect a significant association with T2DM with our relatively small sample size and limited number of incident cases. Based on the observed HR of 1.25 for incident prediabetes in the current study, our power of study to detect a significant association (P<0.05) of the E23K variant with incident prediabetes by the Cox regression analysis was 85.8%, while the power for detecting a significant association with incident T2DM was only 58.1%, even when the same HR was applied in the calculation.

Given the discrepancies in prospective studies regarding the association of the E23K variant with incident T2DM, we tried to resolve this problem by conducting a meta-analysis of reported prospective studies. In the current meta-analysis which involved a total of 15680 subjects across different ethnic groups including our own, we did observe a significant association of the E23K variant with incident T2DM. This has clearly demonstrated that a large sample size is crucial for detecting a genetic variant with modest effect size. One limitation of the current meta-analysis was the difference in diagnostic criteria for T2DM in different studies which might lead to problems in the grouping of subjects. However, due to the limited number of available prospective studies on the E23K variant, we were unable to perform subgroup analyses.

Functional studies have suggested that the E23K variant may affect insulin secretion [7], [8]. Examining the indices of insulin secretion would help to elucidate the possible effect of *KCNJ11* on insulin secretion. However, we were unable to detect any significant association of the E23K variant with HOMA-beta. In fact, previous studies also failed to show the association of this variant with HOMA-beta [4], [5], [25], [26]. Being a simple way to estimate insulin secretion, HOMA-beta may not be the best index of insulin secretion since its derivation relies solely on the basal glucose and insulin levels. Other ways of estimating insulin secretion that provide more optimal assessments of beta-cell function, for example, the acute insulin responses during oral or intravenous glucose tolerance test, or the disposition index would be preferred. Unfortunately, the limited data available in our study did not allow us to carry out such analyses.

In conclusion, we had demonstrated that the *KCNJ11* E23K variant independently increased the risk of developing prediabetes in this prospective study, supporting its role in glycaemic progression in Southern Chinese, with the effect being more evident at the stage when NGT subjects progressed to prediabetes. We were unable to demonstrate a significant association of this genetic variant with T2DM, likely due to our relatively small sample size. In the meta-analysis in which our data were combined with those from seven other reported prospective studies, the E23K variant was indeed shown to be predictive of T2DM.
